# The Role of the RNA Helicase DDX3X in Medulloblastoma Progression

**DOI:** 10.3390/biom14070803

**Published:** 2024-07-06

**Authors:** Akanksha Swarup, Timothy A. Bolger

**Affiliations:** Department of Biochemistry and Molecular Biology, University of Georgia, Athens, GA 30602, USA

**Keywords:** medulloblastoma, DDX3X, RNA helicase, translation

## Abstract

Medulloblastoma is the most common pediatric brain cancer, with about five cases per million in the pediatric population. Current treatment strategies have a 5-year survival rate of 70% or more but frequently lead to long-term neurocognitive defects, and recurrence is relatively high. Genomic sequencing of medulloblastoma patients has shown that *DDX3X*, which encodes an RNA helicase involved in the process of translation initiation, is among the most commonly mutated genes in medulloblastoma. The identified mutations are 42 single-point amino acid substitutions and are mostly not complete loss-of-function mutations. The pathological mechanism of *DDX3X* mutations in the causation of medulloblastoma is poorly understood, but several studies have examined their role in promoting cancer progression. This review first discusses the known roles of DDX3X and its yeast ortholog Ded1 in translation initiation, cellular stress responses, viral replication, innate immunity, inflammatory programmed cell death, Wnt signaling, and brain development. It then examines our current understanding of the oncogenic mechanism of the *DDX3X* mutations in medulloblastoma, including the effect of these *DDX3X* mutations on growth, biochemical functions, translation, and stress responses. Further research on DDX3X’s mechanism and targets is required to therapeutically target DDX3X and/or its downstream effects in medulloblastoma progression.

## 1. Introduction

Medulloblastoma is a central nervous system tumor that accounts for 20% of childhood brain tumors and 7–8% of all brain tumors [1]. There are around 400 new cases diagnosed in the United States annually. It is described as a “malignant invasive tumor of the cerebellum of the cerebellum with preferential manifestation in children, and an inherent tendency to metastasize via cerebrospinal (CSF) pathways” [2]. The World Health Organization describes medulloblastoma as a Grade IV tumor, indicating that it is highly malignant, mitotically active and necrosis-prone [2].

Medulloblastomas are the most common malignant pediatric tumors in the brain. They were first identified by Bailey and Cushing in 1925 [3]. On a computed tomography (CT) scan, a medulloblastoma presents as a hyperdense mass in the cerebellum [3]. Symptoms of medulloblastoma present as cerebellar dysfunction and increased intracranial pressure (ICP) since the cancer arises in the fourth ventricle, and this often results in hydrocephalus [3,4]. ICP manifests in patients physically as irritability, lethargy, nausea and vomiting, morning headaches, anorexia, and behavioral changes. Cerebellar dysfunction is displayed as truncal ataxia (impaired tandem gait) [3]. Medulloblastoma is initially often misdiagnosed or diagnosed late in children since the initial symptoms, such as gastroenteritis, headaches, vertigo, irritability, poor feeding and loss of milestones, are not specific to medulloblastoma [3,4]. This leads to an unfortunate delay in diagnosis and subsequent neurocognitive impairment, even if treatment has been provided on time and the cancer has been brought under control. Hence, there is a need to ensure neurologic examinations for children exhibiting these symptoms. Currently, the initial diagnosis test for medulloblastoma is brain imaging via a head CT or brain magnetic resonance imaging (MRI) [4].

Pre-treatment prognosis of medulloblastoma was traditionally associated with its histopathologic classification as follows: large-cell medulloblastoma, anaplastic medulloblastoma, desmoplastic/nodular medulloblastoma and medulloblastoma with extensive nodularity [4]. However, another method of classification of the medulloblastoma has been described based on molecular sub-types designated via transcriptional profiling studies [5]. Four molecular sub-types are designated by the affected signaling pathway. They are as follows: Wingless (WNT), Sonic Hedgehog (SHH), Group 3 and Group 4. These sub-types have proven to be fairly representative of the prognosis and can be used for risk stratification [3,4]. For example, the WNT subtype has the best prognosis overall, with a 5-year survival rate of about 95%, while Group 3 has the worst, with a 5-year survival rate of only 50% [6]. However, currently, there is no difference in treatment provided to patients with different molecular subtypes of medulloblastoma.

Medulloblastoma manifests differently in adults versus in children, and different treatment strategies are required for both [7]. There are clinical differences (adult medulloblastomas are not metastatic at diagnosis) as well as histological differences between adult and pediatric medulloblastoma. Since the disease is mostly prevalent in children, it is mostly researched in children, while studies and clinical trials for adult medulloblastomas are lacking. Adult patients also do not show the favorable chances of survival that children do when diagnosed with the WNT subtype [7].

Historically, the efficacy of medulloblastoma treatment, recovery, and quality of life post-treatment has been tenuous at best. In the 1950s, it was dismal, with surgery being the only treatment for these tumors at the time. Eventually, medical advances in radiation methods such as craniospinal irradiation made it possible for the 3-year survival to be 65%. However, this increase in lifespan was accompanied by neurocognitive impairment, secondary malignancies, and endocrine dysfunction. Starting in the 1970s, radiation techniques were accompanied by chemotherapy and surgery, which both improved survival outcomes and allowed a decrease in radiation doses. At the current time, standard treatment still includes a combination of surgery, radiation, and chemotherapy. Common chemotherapeutic strategies include using either a combination of cisplatin, lomustine, and vincristine or a combination of cisplatin, cyclophosphamide, and vincristine. The five-year survival rate of the first strategy is around 81% and that for the second one is 86% [4]. Current therapeutic strategies possibly over-treat children with the WNT subtype, which is dangerous and leads to long term neurocognitive defects [8]. Furthermore, radiation and broad-based chemotherapies often cause later developmental and neurocognitive defects even if patients are not over-treated [4].

There is, however, promise of developing new targeted therapies for this disease by combining the molecular subgroupings with the results of deep sequencing studies that identified additional potential driver mutations in certain genes. One of these genes is *DDX3X*, which undergoes single point mutations and encodes an RNA helicase involved primarily in translation initiation [6,8,9,10,11]. Genomic sequencing of medulloblastoma patients provided data supporting the involvement of these *DDX3X* mutations in medulloblastoma occurrence, and several studies, discussed below, have subsequently examined their potential role in promoting cancer progression. Further work is required, however. Understanding the pathological mechanism of the *DDX3X* mutations will lead to a better understanding of the course of medulloblastoma and may provide an additional biomarker for diagnosis. Additionally, as an enzyme, DDX3X is potentially a specific target for therapeutics, which could decrease the long-term cognitive impairment and improve quality of life post-treatment for many patients.

### 1.1. Introduction to DDX3X

#### 1.1.1. The DEAD-Box RNA Helicase Family

DDX3X belongs to Superfamily 2 (SF2) of RNA helicase proteins and more specifically to the DEAD (Asp-Glu-Ala-Asp)-box family [12,13]. Proteins belonging to the DEAD-box family have a helicase core, conserved from prokaryotes to humans, that consists of two domains—the D1 and D2 RecA-like domains that contain 12 highly conserved sequence motifs. These conserved motifs, including the eponymous DEAD sequence, mediate RNA and nucleotide binding [14]. The conserved core is required for the helicase activity of the protein and is flanked by N- and C-terminal extensions on either side of the helicase domains that mediate regulatory and protein–protein interactions [12,15]. DEAD-box proteins (DBPs) are critical for many RNA-based cellular processes, including most steps of gene expression, ribosome biogenesis, and others.

The DBP family of proteins carry out their helicase activity in a cycle of RNA and nucleotide binding [12,16]. The DBP cooperatively binds adenosine triphosphate (ATP) and double-stranded RNA (dsRNA), and this binding induces a “closed” conformation of the RecA-like domains. This conformation then forces the local unwinding of the RNA duplex by excluding one RNA strand. ATP hydrolysis is also stimulated by this conformation, which promotes release of the RNA from this complex. The RecA-like domains then assume an “open” conformation, and the cycle can restart. Some studies have suggested that DBPs can act cooperatively as dimers or trimers in unwinding, but whether this is the case in vivo is unclear [15,17,18]. Functionally, DBPs often serve as “RNA/RNP chaperones”, meaning that they assist in RNA folding to form appropriate secondary structures and to regulate ribonucleoprotein complex dynamics. Due to their high propensity for intramolecular interactions, RNA molecules involved in many cellular processes need such chaperones to assist in structural transitions and efficient folding, as well as to regulate the multitude of RNA–protein interactions [12,14]. Although DBPs appear to have specific roles in different processes, most lack inherent RNA sequence specificity and can bind to a variety of mRNAs since the helicase core binds to the sugar–phosphate backbone of the RNA [13]. Specificity is therefore thought to derive from protein–protein interactions, often with the N- and C-terminal extensions [19].

#### 1.1.2. DDX3X and Translation Initiation

**Figure 1:**
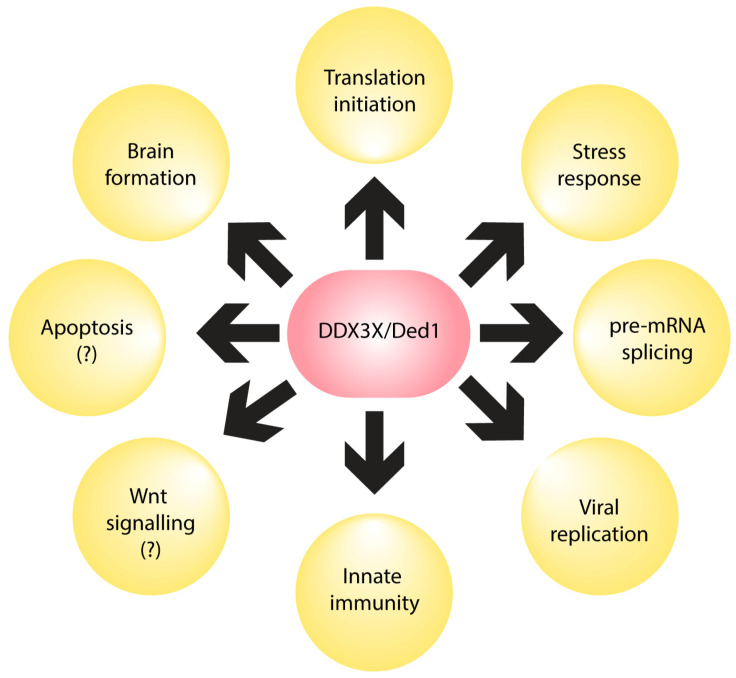
Functions of DDX3X. Schematic to demonstrate the various processes in which DDX3X is involved at the cellular and organismal level. These processes include translation initiation [20,21,22,23,24,25,26,27,28,29,30], stress response [23,28,31,32,33,34,35,36,37,38,39,40], pre-mRNA splicing [20,41], viral replication [42,43,44,45,46,47,48], innate immunity [49,50,51,52,53,54,55], Wnt signaling [10,56], apoptosis [57,58] and brain formation [59,60,61,62,63,64,65,66,67,68,69]. Question marks indicate that the role of DDX3X in these processes requires further study.

The protein DDX3X and its yeast ortholog Ded1 are primarily associated with translation initiation, although they may also have other roles, as described below in other subsections (Figure 1) [20,21]. In translation, mutations in catalytically critical amino acid residues in *DED1* have been shown to result in the rapid accumulation of non-translating 80S ribosomes and a reduction in polysome formation, which implies a role for DDX3X/Ded1 in overall protein synthesis [20]. The canonical role for DDX3X/Ded1 in translation is to promote start site scanning through the 5′ untranslated region (UTR) [22,23,24,25].

Specifically, DDX3X/Ded1 is recruited to the eukaryotic initiation factor 4F (eIF4F) complex at the 5′ end of target mRNA, where it unwinds 5′UTR stem-loop structures in the mRNA and allows the 43S pre-initiation complex (PIC) to scan the mRNA for the AUG start codon [24,25]. Studies carried out by Guenther et al. show that impaired Ded1 activity in yeast results in the stabilization of RNA structures in the 5′ UTR and activation of near-cognate alternative translation initiation sites (ATIS), which in turn can either stimulate or inhibit the translation of the main coding open reading frame (ORF). This implies that Ded1 in yeast is involved in deciding the location of translation initiation on the mRNA—whether at the primary AUG, at near cognates, or possibly at small upstream ORFs (uORFs) [26]. In addition, Ded1 also plays a role in pre-initiation complex assembly [27,28]. Ded1 binds to several members of the eIF4F complex, including the scaffolding factor eIF4G1, the cap-binding protein eIF4E, and fellow DBP eIF4A, and it promotes eIF4F loading onto the mRNA, possibly in an mRNA-specific manner [18,28,29,30]. Although less well characterized, mammalian DDX3X appears to function in much the same manner [20]. Thus, DDX3X/Ded1 can potentially influence translation in complex ways through its effects on different initiation processes and on different mRNAs. 

#### 1.1.3. DDX3X and the Stress Response

Despite its normal function in promoting translation initiation, some evidence suggests that DDX3X/Ded1 may also function as a translational repressor [28,31]. Specifically, work by several different groups has converged over time to show important roles for DDX3X/Ded1 during the cellular response to stress conditions. First, both DDX3X and Ded1 are associated with stress granules (SGs), large cytoplasmic RNP foci that are formed in response to environmental stressors [23,28]. SG formation, which is often stimulated by the liquid–liquid phase separation of intrinsically disordered protein domains, is closely associated with translation repression, although the causality between these processes has been a matter of debate [32,33,34]. Manipulation of DDX3X/Ded1 activity or levels has been shown to affect SG assembly in yeast and humans [28,31,35,36]. 

These effects likely derive from interactions in the N- and C-terminal regions (which are intrinsically disordered regions in DDX3X/Ded1), and they have been shown to drive the phase separation of DDX3X/Ded1 in vitro [37]. Furthermore, Iserman et al. showed that heat stress also results in the phase separation of recombinant Ded1, and in cells, Ded1-containing RNP condensates are formed during heat shock that resemble SGs [38]. The authors proposed that Ded1 sequestration in these condensates reduces the translation of “housekeeping” mRNAs compared with that of stress response mRNAs as an adaptive response under severe heat stress.

As another stress-dependent function, yeast Ded1 has also been shown to directly regulate translation under stress conditions. Aryanpur et al. observed that when cells are treated with the TORC pathway inhibitor rapamycin, eIF4G1 is dissociated from ribosomal complexes by Ded1, leading to its degradation [39]. The authors suggested a model where under conditions of cellular stress, Ded1 stalls translation and removes eIF4G1 from the PIC, thus decreasing global translation. This model is consistent with the rapid dissociation of Ded1 and the eIF4F complex from mRNA observed by Bresson et al. during glucose deprivation [40]. To begin to develop an overall model of the stress function of DDX3X/Ded1, a further study by Aryanpur and colleagues discussed a two-phase response to cellular stress: a stress response phase and an adaptation/recovery phase, which each included effects on growth, translation, and stress granules. They proposed that Ded1 may play roles in both translation and SGs (and thus growth) during both phases [36].

#### 1.1.4. Non-Translational Functions of DDX3X

DDX3X/Ded1 may also play roles outside of translation, although in some cases the ascribed function may be an indirect effect of its role in translation. In yeast, Ded1 was initially linked to pre-mRNA splicing, primarily as a suppressor for mutations in the splicing factor PRP8 [41]. However, little follow-up has been carried out, and the exact role of Ded1 in splicing remains to be clarified [20]. Other ‘moonlighting’ functions of DDX3X/Ded1 are discussed below. 

Human DDX3X is known to be an important factor in the replication of several different viruses, including HIV-1 and HCV, and the protein is under study as a target for antiviral therapy and treatment [42]. DDX3X function appears to be hijacked in multiple ways by different viruses to promote infection and replication. For HIV, DDX3X has been shown to promote both the nuclear export of HIV-1 RNA and translation of the RNA via its direct interaction with HIV1-1 Tat protein [43,44]. Likewise, DDX3X may play multiple roles in HCV replication, including associating with HCV RNA in lipid droplets that serve as viral replication and packaging sites [45,46]. Thus, DDX3X inhibitors have been of interest as antivirals, and recently, DDX3 inhibitors were shown to reverse HIV-1 latency in viral RNA-expressing CD4+ T cells [47,48]. 

In addition to its pro-viral role, DDX3X is also involved in innate anti-viral immunity, in particular contributing to Type I interferon production at multiple levels [50]. Among the most well-characterized interactions with immune system components is its role in the RIG-I sensing pathway for viral dsRNAs, in which DDX3X is stimulated to act as a scaffolding adapter to IκB kinase ε. This in turn enhances the kinase’s phosphorylation of the transcription factor IRF3 and IFNβ promoter induction [49,51]. DDX3X plays a role in IKKα activation and its downstream signaling pathways as well [52]. Furthermore, DDX3X has also been reported to act upstream as a viral sensor, binding to RNAs as well as the mitochondrial antiviral-signaling protein (MAVS). DDX3X’s role in IFNβ induction was also shown to be suppressed by the HCV core protein [53]. DDX3X may be essential for hematopoiesis and immune cell maintenance as well as signaling [54]. Finally, DDX3X may promote a form of inflammatory programmed cell death, pyroptosis, through the activation of the sensory protein NLRP3 in caspase-1-containing inflammasomes. Samir et al. proposed a model wherein SGs have a cell-protective effect by competing for the association of DDX3X with NLRP3 and inflammasomes, turning DDX3X into a “live-or-die checkpoint” in inflammation, although how DDX3X localization is regulated in these decisions is not clear [55]. 

DDX3X has also been linked to other signaling pathways as well. Most relevant for its potential functions in medulloblastoma, DDX3X has been reported to play a role in Wnt signaling [10,56]. Canonical Wnt signaling stabilizes the transcription factor ß-catenin due to the inhibition of the destruction complex by the receptor complex and Dishevelled (Dvl) [53]. In this pathway, DDX3X is a regulatory subunit of casein kinase 1 ε (CK1ε) that binds to it and stimulates its kinase activity [56]. This in turn results in the phosphorylation of Dvl and assembly of the WNT/β-catenin signalosome. DDX3X is also implicated in the upregulation of DNA damage-induced apoptosis regulation and p53 accumulation in the nucleus, with Sun et al. reporting that there may be a direct association between the two as a result of co-immunoprecipitation experiments [57]. DDX3X was found to associate with p53 during the process of mitosis as well, where it localizes to the centrosome and co-localizes with centrosome-associated p53 [58].

#### 1.1.5. DDX3X in Brain Development

Fittingly for a protein implicated in brain cancer, DDX3X is important for normal function in brain development as well. *Ddx3x* expression is ubiquitous in mouse embryos but is especially enriched in the developing brain, including the cortex and hippocampus [59]. Expression has been observed in both neurons and other cell types in the brain [59]. Germline knockouts of *Ddx3x* in mice cause early post-implantation lethality [60], but conditional knockouts and transient sgRNA-mediated knockdowns have been used to examine its role in brain development [59,61,62]. Neural-specific knockout mice have overall microcephaly, including reduced cortex size, and they have progressive disorganization and loss of structure in the developing cerebellum [61,62]. These effects appear to be due primarily to defects in neuron differentiation and possibly neural stem cell proliferation [59,62]. In cell culture models, *Ddx3x* knockdown also reduced neurite outgrowth [63], although little effect was observed in post-mitotic neurons in vivo [62]. Interestingly, *DDX3Y*, a *DDX3X* paralog located on the Y chromosome in both humans and mice, appears to be able to compensate for the loss of *Ddx3x* in the brains of male mice, such that the phenotypes of *Ddx3x* hemizygous male mice and heterozygous female mice are similar [61,62]. The mechanism by which *Ddx3x* affects brain development has not been fully defined, although Hox gene downregulation has been observed in conditional knockouts [61]. Additionally, a recent study by Hoye et al. conducted ribosomal profiling in the cortexes of knockouts and identified a subset of affected mRNAs, of which some affect neurogenesis [62].

DDX3X has also been implicated in brain development in humans as well. Mutations in *DDX3X* can cause DDX3X Syndrome, a cognitive disorder that was first described in 2015 [64]. DDX3X Syndrome affects mostly females and has a range of symptoms, including developmental delays, intellectual disability, behavior problems, movement disorders, and facial abnormalities [65]. Similarly, DDX3X is also associated with autism spectrum disorder [66,67]. A behavioral study using heterozygous *Ddx3x* mice has shown various physical, sensory, and motor deficiencies that are similar to DDX3X Syndrome symptoms, suggesting that the syndrome-associated mutations are likely to be loss-of-function [68]. Transient overexpression of these mutations in cell lines induces SGs and has specific effects on translation [59]. Interestingly, while a few of the reported mutations in DDX3X Syndrome overlap with those identified in medulloblastoma, for the most part, they are distinct and include multiple types of mutations (e.g., nonsense) not found in medulloblastoma [64,69].

## 2. DDX3X Mutations and Medulloblastoma

In 2012, a series of papers were published that used next-generation sequencing techniques to focus on the genomic changes associated with pediatric medulloblastoma, and they identified *DDX3X* as one of the commonly recurring mutated genes in this disease [8,9,10]. Jones et al. performed deep-sequencing analysis studies and identified several recurrent mutations in already-known medulloblastoma-related genes such as *CTNNB1*, *PTCH1*, *MLL2*, and *SMARCA4*, as well as a new set of genes not linked to this tumor before; this set included *DDX3X*, *CTDNEP1*, *KDM6A*, and *TBR1* [9]. Pugh et al. found that the *DDX3X* mutations often occurred concurrently with mutations in *CTNNB1*, the gene for β-catenin. Robinson et al. identified 41 novel genes with recurrent somatic mutations in medulloblastoma in the context of the different subgroups, including *DDX3X*. Overall, *DDX3X* was the second most frequently mutated gene in these studies after *CTNNB1* [6]. *DDX3X* mutations were most prevalent in the WNT subgroup, where they were found in 50% of cases, but mutations were found in other subtypes as well. In particular, *DDX3X* was also mutated in the SHH subtype in about 11% of pediatric cases [6], and in a later study by Kool and colleagues, *DDX3X* mutations were found in over half of adult cases of the SHH subtype [11]. These results were echoed in 2020 when Wong et al. showed that the most frequently mutated genes in adult medulloblastoma are *TERT*, chromatin modifiers *KMT2D* and *KMT2C*, *TCF4*, *PTCH1*, and *DDX3X* [7].

**Figure 2:**
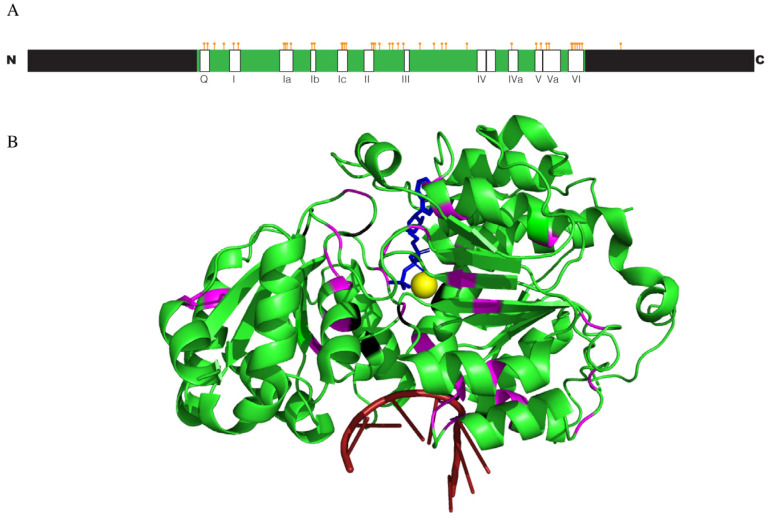
Location of the medulloblastoma-associated mutations in DDX3X. (**A**) A sequence schematic of DDX3X with the helicase core in green and the N- and C-terminal regions in black. The identified mutations (orange lines) and conserved DEAD-box helicase motifs (labeled in rectangles) are marked. (**B**) Crystal structure of DDX3X from (PDB ID: 7LIU) [71] with medulloblastoma-associated mutations marked. Sites that were lethal in yeast Ded1 are shown in black while the rest are shown in pink [70]. An ATP analog is shown in blue, single-stranded RNA is shown in red, and magnesium ion is shown in yellow.

All in all, the sequencing studies identified 42 different sites in *DDX3X* that were mutated in medulloblastoma patients (Table 1). Most of these were single missense mutations, although three in-frame, single-amino-acid indels (two deletions and one insertion) were also found. The mutations were found to be spread across the conserved helicase domain of the protein (Figure 2A,B), but there seems to be no obvious unifying structural or functional motif affected by these mutations [70]. Based on their putative location in the DDX3X protein structure, Robinson speculated that these mutations interfered with nucleic acid binding and may alter specificity and/or affinity for RNA substrates [8]. However, looking at the broader spectrum of where the mutations are located, this may not be true (see the biochemical section below). Overall, the sequencing results suggest that these missense mutations have a specific effect on DDX3X function and do not represent a straightforward loss-of-function phenotype.

### 2.1. Oncogenic Mechanism of DDX3X Mutations

Several studies have examined subsets of the medulloblastoma-associated *DDX3X* mutations in greater detail and documented various biochemical, molecular, and cellular defects associated with them (Table 1, Comments column), suggesting that these phenotypes contribute to the role of DDX3X in medulloblastoma. However, in most cases, it is unclear whether a particular phenotype is especially critical, especially given that the large number of mutation sites could result in varied phenotypes. Furthermore, it remains possible that the mutations could promote cancer progression in more than one way or that different mutations have different contributions. Here, we will discuss the current state of knowledge of the effects of the *DDX3X* mutations, organized by phenotype: growth, biochemical defects, translational defects, stress granule formation, tissue-level effects, and other potential effects.

#### 2.1.1. Effect of Mutations on Growth

Several groups have examined whether the medulloblastoma-associated mutations in *DDX3X* cause growth defects in model organisms such as yeast. Epling et al. studied the role of these DDX3X mutations using *Schizosaccharomyces pombe* as their model [72]. They assessed whether the DDX3X mutants could complement a cold-sensitive *ded1-61* mutant and saw that while wild-type *DDX3X* and some mutants restored growth under restrictive cold temperatures, others did not. These growth differences did not appear to result from differences in *DDX3X* expression. They thus divided their mutants into non-complementing (A222P, G302V, G325E, and P568L) and “functional” (complementing) mutants (T275M, R351W, L353F, D354V, and M370R). Interestingly, a mutation in the Walker A motif (K230A, not found in medulloblastoma) that rendered DDX3X completely ATPase-defective had a dominant growth defect, suggesting that none of the medulloblastoma-associated mutations are as severe.

Two other studies have examined growth phenotypes in Saccharomyces cerevisiae, both of which generated mutations in the yeast ortholog *DED1* that correspond to the medulloblastoma-associated ones in *DDX3X*, taking advantage of the high degree of identity between *DDX3X* and *DED1* at these sites. Floor et al. assessed the growth of a limited set of mutants, and similar to the results in *S. pombe*, they observed that while some *ded1* mutations (R235A and R235K) were tolerated, others (R235Y, R492A, and R492H) were inviable [74]. Brown et al. conducted a more extensive analysis, examining the growth of mutants in all 42 different mutation sites at temperatures of 16 °C, 25 °C , 30 °C , and 37 °C [70]. Only nine of the 42 mutations were lethal at all four temperatures. Of the remaining 33 mutants, 19 displayed temperature-sensitive growth (16 being cold-sensitive and 3 being heat-sensitive). Thus, two-thirds (28 out of 42) of these mutants showed growth defects in this study, and the results largely agree with the earlier studies for specific mutants.

Overall, the growth assays show that the medulloblastoma-associated mutations have significant phenotypes in yeast. These results suggest that the medulloblastoma-associated mutations affect the function of DDX3X but are mostly not complete loss-of-function mutations. Furthermore, defects in Ded1 activity in yeast may largely recapitulate the molecular effects of *DDX3X* mutation in medulloblastoma.

#### 2.1.2. Effects on the Biochemical Function of DDX3X 

The three studies above also examined the effect of the medulloblastoma-associated mutations on the biochemical activity of the DDX3X/Ded1 protein in vitro [70,72,74]. First, Epling and colleagues modeled the potential structural changes in DDX3X induced by a subset of the mutations. No large-scale changes were observed, but they did predict RNA binding and/or ATP hydrolysis defects for a few mutants. They then examined ATP hydrolysis and RNA binding in two of each of their functional and non-complementing mutants [72]. Both non-complementing mutants (G302V and G325E) displayed severe defects in ATP hydrolysis, while one functional mutant, L353F, had only a minor defect, and another (D354V) had no defect in ATPase activity. Interestingly, only G302V had a significant defect in RNA binding, even though the G325E mutation is located in a motif required for RNA binding (Table 1). These results indicate that at least some of the DDX3X medulloblastoma mutants have significant defects in biochemical activity, but they are variable between different mutants. Consistent with this interpretation, Floor et al. also showed substantial but variable defects in duplex unwinding for the R276 and R534 mutants of DDX3X that largely correlated with the growth phenotypes observed in ded1 mutants [74].

Brown and colleagues also examined in vitro ATP hydrolysis activity and RNA binding for 11 additional mutants of yeast Ded1 [70]. Several of the mutants showed reduced ATPase activity, ranging from mild (R285C) to severe (S371F), while others did not have a significant defect. Likewise, only three mutants (T166A, M339I, and G488A) displayed significant RNA binding defects, suggesting that other biochemical activities besides RNA binding are likely to be affected in the other mutants. Furthermore, the observed defects did not correlate well with the growth defects of these mutants, suggesting that there may be multiple permutations of biochemical defects that contribute to medulloblastoma pathology. Moreover, the variety of defects in the different mutants implies that these mutations do not promote medulloblastoma pathology via a common biochemical activity. Rather it is likely that a variety of biochemical changes cause similar alterations in DDX3X’s function at the cellular level instead.

#### 2.1.3. Effect on Translation

Given that the primary function of DDX3X is in translation, it has been speculated that the medulloblastoma-associated *DDX3X* mutations may impair global translation [72,73]. Consistent with this idea, Epling et al. showed that protein levels of a known Ded1 target were reduced in several medulloblastoma-associated *DDX3X* mutants in *S. pombe* [72]. More directly, Valentin-Vega et al., using HEK293T cells with overexpressed *DDX3X*, showed that several mutants (G302V, G325E, and M370R) inhibited general translation as measured by 35S-Met labeling and puromycin incorporation [73]. Likewise, ribosomal profiling (Ribo-seq) of one mutant (G325E) showed a broad reduction in footprint density across the coding regions of mRNAs, consistent with a general defect. However, Brown et al. generated polyribosome profiles from a larger subset of the medulloblastoma-associated mutants in DED1, and while some mutants had significant defects in this assay, others did not [70]. Furthermore, they found that the global translation defects correlated poorly with the cell growth phenotypes. This implies that the medulloblastoma mutations do not simply and straightforwardly reduce global translation but are more likely to affect the translation of critical gene subsets. Indeed, it seems unlikely that global translation inhibition would promote cancer, although Valentin-Vega et al. suggest that such inhibition may be a part of metabolic adaptation to nutrient deprivation [73].

Efforts to identify the critical target mRNAs of DDX3X in medulloblastoma have not yet conclusively defined such a subset. In 2016, Oh and colleagues analyzed mRNA levels in SHH-subtype medulloblastoma patients, and via gene set enrichment analyses, they showed that the mRNAs of genes correlated with translation initiation had elevated levels in tumors with wild-type *DDX3X* compared with those of *DDX3X*-mutant tumors [75]. Given that mRNA levels rather than translation were examined, however, these are indirect targets of DDX3X activity. To attempt to identify direct targets, both Valentin-Vega et al. and Oh et al. performed CLIP-seq with wild-type DDX3X. The former showed enrichment for genes involved in RNA metabolism, while the latter showed binding to a large fraction of the transcriptome overall [73,75].Valentin-Vega et al. also showed that translation of a small subset of the identified targets was impaired when the G302V and G325E mutants were overexpressed [73]. In contrast, Oh et al. did not observe a substantial effect on translation in normal conditions when a different mutant (R534H) was overexpressed in HEK293 cells [75]. However, changes in both translation (by Ribo-seq) and in DDX3X binding to mRNAs were observed in this mutant in the presence of sodium arsenite, a stressor known to induce SGs. Interestingly, arsenite-insensitive mRNAs were enriched for genes involved in chromatin assembly, and showed increased binding to the R534H mutant, while arsenite-sensitive mRNAs were enriched for genes involved in protein transport and localization in wild-type *DDX3X*-expressing cells, and these were less enriched in the mutant cells (also see the next section). How these potential DDX3X targets are regulated is not clear; however, Brown et al. compared the relative translation of luciferase reporters with structured and unstructured 5′ UTRs, respectively, in the medulloblastoma-associated *ded1* mutants in yeast and found that virtually all the mutants had defects in translating mRNAs with more structured 5′ UTRs [70]. Taken together, these results suggest that the medulloblastoma mutations in *DDX3X* induce complex changes in the translatome, likely through the effects of DDX3X on 5′ UTR structure, resulting in medulloblastoma progression. Further research is needed to better describe these changes.

#### 2.1.4. Effect on the Stress Function of DDX3X

Because tumor cells are frequently under stress conditions, and stress responses are often dysregulated in cancer, the role of DDX3X in cellular stress may be impacted by the medulloblastoma-associated mutations. Valentin-Vega and colleagues observed a significant upregulation in SG formation upon the overexpression of several DDX3X mutants in both HEK293T and HeLa cells [73]. SG hyper-assembly in the mutants could also be reversed by deleting the N-terminal low-complexity domain in DDX3X or by knocking down SG assembly factors G3BP1/G3BP2. Likewise, Brown et al. showed a similar increase in constitutive SGs in the yeast *ded1* mutants, and Oh et al. observed a moderate increase in SGs in the R534H mutant [70,75]. The implications of these effects on SGs are not completely clear, although Valentin-Vega et al. reported an increase in translation when SG formation was blocked by G3BP knockdown [73]. It is also possible that the mutations directly affect the translational function of DDX3X during stress, similar to the role described above for Ded1 in repressing translation during stress [28,36]. Consistent with this idea, in HEK293 cells under arsenite stress, while overexpressed wild-type *DDX3X* resulted in downregulation of translation beyond that caused by the stress itself, this repression was attenuated in cells overexpressing the R534H mutant [75]. In addition, preliminary data from our lab indicate that most of the ded1-mam mutants have rapamycin-resistant growth phenotypes (A.S., unpublished observation). These results imply that DDX3X’s role in stress responses might be affected due to these mutations, which may play a role in medulloblastoma by allowing cancer cells to evade stress- or nutrient-induced growth inhibition.

#### 2.1.5. Other Potential Effects of DDX3X Mutations in Medulloblastoma

Although most studies of DDX3X have focused on its most well-known roles in translation, the medulloblastoma-associated mutations may affect a different function (see non-translational functions of DDX3X above). Such effects are also not necessarily mutually exclusive with effects on translation. The most obvious potential connection is the ability of DDX3X to stimulate CK1ε activity, which in turn activates Wnt signaling. Pugh et al. tested the ability of overexpressed wild-type *DDX3X* and a subset of mutants to activate a WNT-responsive reporter and observed mild activation with a few of the mutants [10]. Several of the *DDX3X* mutants also showed a minor effect on cell viability in conjunction with the overexpression of mutant β-catenin [10]. To our knowledge, however, there are no reports of whether the mutations affect DDX3X binding or the activation of CK1ε. It is also possible that another function of DDX3X, such as its role in innate immunity, may be affected by the medulloblastoma mutations, but such phenotypes have not been reported either.

#### 2.1.6. Effects of DDX3X on Medulloblastoma in Mouse Models

On a tissue level, DDX3X may play a role in maintaining the lineage of rhombic lip progenitor cells (LRLPs), the primary source of WNT-subgroup medulloblastomas [8]. Robinson et al. showed this by performing experiments on mouse hindbrains and observed that, upon knocking down *Ddx3x*, the self-renewal rate of LRLPs was halved. They performed in utero migration assays and showed that mice electroporated with Ddx3x mutants T275M and G325E showed increased proliferation of LRLPs [8].

More recently, Patmore et al. examined the effects of *Ddx3x* knockouts in mice genetically predisposed to WNT or SHH medulloblastoma [61]. Tumors in both models were more broadly distributed (to both the cerebellum and brainstem) in *Ddx3x* knockout lines, suggesting that functional DDX3X restricts the cell lineage origins of medulloblastoma. In cerebellar samples, upregulated Wnt signaling led to the activation of stress response and cell death genes, as measured via RNA levels, and *Ddx3x* knockout reduced these effects, suggesting that loss of DDX3X function may relieve the “oncogenic stress” of increased Wnt signaling. A similar though more moderate effect was observed in the SHH model. Patmore et al. also performed single-cell RNA-seq of a small cohort of WNT subgroup human patient samples, comparing mutant and wild-type *Ddx3x*. Their results showed that RNA levels of genes associated with stress granules, stress response, cell death, and DNA repair were altered in *Ddx3x* mutant tumors, similar to their findings in the mouse model. The authors suggest that normally, DDX3X may function to promote cell death through pyroptosis upon increased Wnt or Shh signaling in certain brain tissue lineages (but perhaps not others). Loss of DDX3X would thus reduce cell death and promote WNT- and/or SHH-dependent oncogenesis in these lineages, suggesting that DDX3X is a tumor suppressor [61]. As a cautionary note, however, these experiments were performed with *Ddx3x* knockouts rather than any of the identified mutations, so these observed effects may be different in actual medulloblastoma patients. Furthermore, this analysis primarily characterized mRNA levels rather than translation, so the observed effects of DDX3X are presumably indirect. Further research is required to fully characterize the effect of the *DDX3X* medulloblastoma mutations in vivo, perhaps including the generation of *Ddx3x* mutant knock-in mouse lines.

## 3. Conclusions and Future Directions 

As we discussed in this review, years of studying the RNA helicase DDX3X have given us a plethora of information on their biological functions, but despite the correlation between its mutation and medulloblastoma progression, more research remains to be conducted in order to link DDX3X function and cancer. DDX3X and its yeast ortholog Ded1 have functions related to eukaryotic translation initiation, stress response, immune response and brain development, and in this review, we focused on the dysregulation of these functions in the context of cancer. As multiple studies discussed in this review have shown, *DDX3X* is one of the most commonly recurring mutated genes in medulloblastoma cases, primarily seen in the WNT and SHH-subtypes of medulloblastoma. A substantial number of point mutations have been identified, and these need to be studied in greater detail since these are mostly not complete loss-of-function mutations. These mutations have been shown to alter DDX3X’s response to growth, biochemical defects, translational defects, stress granule formation and tissue-level effects with respect to brain development. Future directions for this work could involve using mouse models with the mutations introduced via CRISPR. While studies on some *DDX3X* mutations in mammalian cells do exist, they are overexpression studies that may not replicate all of the effects of *DDX3X* mutations [73]. 

For improving medulloblastoma treatment, further studying the *DDX3X* mutations could be immensely helpful in identifying the likely prognosis of the patient and possibly provide more targeted therapy. DDX3X inhibitors do exist, and studies have been performed demonstrating their possible effectiveness in a mouse xenograft model [76]. However, it is not clear whether this is the right approach; indeed, several of the published studies suggest that DDX3X is a tumor suppressor rather than an oncogene. An alternative treatment strategy would thus be to restore the wild-type function of mutated *DDX3X* in medulloblastoma patients. However, without a viable method of gene therapy, such a treatment would require further understanding of the mechanism and targets of DDX3X function and how they are affected by the mutations, in order to therapeutically target the factors and pathways most likely to be effective in treating this cancer.

## Figures and Tables

**Table 1:** Characterization of medulloblastoma-associated *DDX3X* mutations and their corresponding mutations in yeast ortholog *DED1*.

*DDX3X* Mutation	Amino Acid Substitution	DEAD-Box Motif	Function	*DED1*	Comments	References
**204**	T/A	Q	ATP-binding	166	Slight cold-sensitive (cs) growth defect, slight defects in translation, defects in 5′ UTR scanning, slight increase in stress granules (SG) (yeast). Slight defects in ATPase activity, RNA-binding affinity (in vitro).	[11,70]
**206**	V/L	Q	ATP-binding	168	Severe cs, slight ts (thermo-sensitive), severe defects in translation, increase in SGs (yeast).	[11,70]
**214**	I/S,KV	-		V176	Lethal (yeast).	[9,11,70]
**222**	A/P	-		184	Lethal (*DDX3X*), no growth defects (*DED1*) (yeast). Increased SGs (HeLa).	[8,70,72,73]
**227**	G/V	I	ATP-binding	189	No growth defects, no translation defects, slight increase in SGs (yeast).	[11,70]
**231**	T/A	I	ATP-binding	193	Severe cs, slight ts (yeast).	[11,70]
**275**	T/M	Ia	RNA-binding	234	Slight cs, no translation defects, defective 5′ UTR scanning (yeast). Defective ATPase activity (in vitro). Increased SGs (HeLa).	[8,11,70,72,73]
**276**	R/K,T	Ia	RNA-binding	235	Variable growth defects (depending on amino acid substitution), severe defects in translation (yeast). Decreased rate of duplex unwinding (in vitro).	[10,11,70,74]
**277**	E/Q	Ia	RNA-binding	236	No growth defects (yeast).	[11,70]
**281**	Q/H	Ia	RNA-binding	240	Lethal (yeast).	[11,70]
**302**	G/V	Ib	RNA-binding	261	Lethal (yeast). Severely defective in RNA-stimulated ATPase activity, defective in binding dsRNA (in vitro). Increased SGs (HeLa). Decreased global translation (HEK293T).	[8,70,72,73]
**303**	G/V	Ib	RNA-binding	262	Severely cold-sensitive, slight ts (yeast).	[11,70]
**325**	G/E,R	Ic	RNA-binding	284	Lethal, defective in RNA-stimulated ATP hydrolysis (yeast). Increased SGs (HeLa). Decreased global translation (HEK293T).	[8,11,70,72,73]
**326**	R/C	Ic	RNA-binding	285	Cs, slight defects in translation, severe defects in 5′ UTR scanning, increased SGs (yeast). Slight defects in ATPase activity, no RNA binding defects (in vitro).	[9,70]
**327**	L/V	Ic	RNA-binding	286	No growth defects (yeast).	[11,70]
**329**	D/V	Ic	RNA-binding	288	No growth defects (yeast).	[9,11,70]
**351**	R/W	II	ATP-binding	310	Cs, slight defects in translation, severe defects in 5′ UTR scanning, increase in SGs (yeast). Slight increase in ATPase activity, no RNA binding defects (in vitro). Shows SGs (HeLa).	[11,70,72,73]
**353**	L/F	II	ATP-binding	312	Slight cs, slight defects in translation, severe defects in 5′ UTR, increased SGs (yeast). Moderate ATPase defect (in vitro). Shows SGs (HeLa).	[8,11,70,72,73]
**354**	D/H,V	-	ATP-binding	313	No growth defects (yeast). No defects in ATPase activity (in vitro).	[8,10,70,72]
**357**	F/S	-		316	Cs, no defects in translation, severe defect in 5′ UTR, slight increase in SGs (yeast). No defect in ATPase activity, no defects in RNA binding (in vitro).	[9,70]
**368**	D/V	-		326	No growth defects (yeast).	[11,70]
**370**	M/R	-		329	No growth defects (yeast). Increased SGs (HeLa). Decreased translation (HEK293T).	[8,70,72,73]
**376**	R/C,S	-		335	No growth defects (yeast).	[9,10,70]
**380**	M/I	-		339	Cs, severe defects in translation, severe defects in 5′ UTR scanning (yeast). No defects in ATPase activity, defective RNA binding (in vitro).	[11,70]
**384**	T/P	III		343	Lethal (yeast).	[11,70]
**393**	A/S,V	-		352	No growth defects (yeast).	[11,70]
**405**	V/L	-		364	Slight cs, no defects in translation, defective 5′ UTR scanning, increased SGs (yeast). No defects in ATPase and RNA binding activity (in vitro).	[11,70]
**412**	S/F	-		371	Cs, no defects in translation, defective 5′ UTR scanning, defective increased SGs (yeast). Defective ATPase activity, no defects in RNA binding (in vitro).	[9,70]
**415**	I/-	-		374	Lethal (yeast).	[11,70]
**434**	L/-	-		393	Slight ts growth defects (yeast).	[11,70]
**475**	R/C	IVa	RNA-binding	433	No growth defects (yeast).	[9,11,70]
**496**	V/M	V	RNA-binding	454	Slight ts (yeast).	[11,70]
**501**	A/-	V	RNA-binding	459	Lethal (yeast).	[11,70]
**504**	G/V	Va		462	Slight cs (yeast).	[11,70]
**506**	D/Y	Va		464	No growth defects (yeast).	[10,70]
**527**	H/Y	VI	ATP-binding	485	Lethal (yeast).	[9,70]
**528**	R/H	VI	ATP-binding	486	Slight cs, defects in 5’ UTR scanning.	[10,11,70]
**530**	G/A	VI	ATP-binding	488	No growth defect (yeast). Defective ATPase, defective RNA-binding activity (in vitro).	[9,70]
**532**	T/M	VI	ATP-binding	490	No growth defect (yeast).	[11,70]
**534**	R/S,H,C	VI	ATP-binding	492	Lethal (yeast). Decreased duplex unwinding (in vitro). Minor defects in translation profile (HEK293T).	[8,9,10,70,74,75]
**536**	G/R	VI	ATP-binding	494	Slight cs, slight defect in translation, defective 5’ UTR scanning, greatly increased SGs (yeast).	[11,70]
**568**	P/L	-		526	Ts, defective 5’ UTR, greatly increased SGs (yeast). Slight defect in ATPase activity (in vitro).	[8,10,11,70,72]

## Data Availability

Not applicable.
